# The influence of drug-induced metabolic enzyme activity inhibition and CYP3A4 gene polymorphism on aumolertinib metabolism

**DOI:** 10.3389/fphar.2024.1392849

**Published:** 2024-05-24

**Authors:** Feng Ye, Jinhuan Ni, Xinyue Li, Jing Wang, Jianchao Luo, Shiyu Wang, Xiaoyu Xu, Yunshan Zhong, Jianchang Qian, Zhongxiang Xiao

**Affiliations:** ^1^ Affiliated Yueqing Hospital, Wenzhou Medical University, Wenzhou, Zhejiang, China; ^2^ Institute of Molecular Toxicology and Pharmacology, School of Pharmaceutical Sciences, Wenzhou Medical University, Wenzhou, Zhejiang, China

**Keywords:** aumolertinib, CYP3A4 gene polymorphism, drug interaction, telmisartan, carvedilol

## Abstract

The purpose of this study is to clarify the drug interaction profile of aumolertinib, and the influence of CYP3A4 genetic polymorphism on aumolertinib metabolic characteristics. Through microsomal enzyme reactions, we screened 153 drugs and identified 15 that significantly inhibited the metabolism of aumolertinib. Among them, telmisartan and carvedilol exhibited potent inhibitory activities in rat liver microsomes (RLM) and human liver microsomes (HLM). *In vivo*, the pharmacokinetic parameters of aumolertinib, including AUC and C_max_, were significantly altered when co-administered with carvedilol, with a notable decrease in the clearance rate CL_z/F_. Interestingly, the pharmacokinetic parameters of the metabolite HAS-719 exhibited a similar trend as aumolertinib when co-administered. Mechanistically, both telmisartan and carvedilol exhibited a mixed-type inhibition on the metabolism of aumolertinib. Additionally, we used a baculovirus-insect cell expression system to prepare 24 recombinant CYP3A4 microsomes and obtained enzymatic kinetic parameters using aumolertinib as a substrate. Enzyme kinetic studies obtained the kinetic parameters of various CYP3A4 variant-mediated metabolism of aumolertinib. Based on the relative clearance rates, CYP3A4.4, 5, 7, 8, 9, 12, 13, 14, 17, 18, 19, 23, 24, 33, and 34 showed significantly lower clearance rates compared to the wild-type. Among the different CYP3A4 variants, the inhibitory potency of telmisartan and carvedilol on the metabolism of aumolertinib also varied. The IC_50_ values of telmisartan and carvedilol in CYP3A4.1 were 6.68 ± 1.76 μM and 0.60 ± 0.25 μM, respectively, whereas in CYP3A4.12, the IC_50_ exceeded 100 μM. Finally, we utilized adeno-associated virus to achieve liver-specific high expression of CYP3A4*1 and CYP3A4*12. In the group with high expression of the less active CYP3A4*12, the magnitude of the drug-drug interaction was significantly attenuated. In conclusion, CYP3A4 genetic polymorphism not only influences the pharmacokinetic characteristics of aumolertinib, but also the inhibitory potency of telmisartan and carvedilol on it.

## 1 Introduction

Lung cancer is one of the most common cancer in terms of incidence and mortality worldwide (Nasim et al., 2019). Non-small cell lung cancer (NSCLC) accounts for the largest proportion, and it has a high mortality rate, with an overall 5-year survival rate of only 15% (Hong et al., 2022). Although there are many targeted therapy drugs for NSCLC currently, drug resistance remains a common challenge that needs to be addressed. Against this backdrop, Aumolertinib (formerly almonertinib; HS-10296) has emerged as a potential solution (Zhang et al., 2021). It is a third-generation epidermal growth factor receptor (EGFR) tyrosine kinase inhibitor that is utilized to treat locally advanced or metastatic NSCLC with T790M EGFR mutation (Wu et al., 2021; Lu et al., 2022a; Ao et al., 2022; Lu et al., 2022b; Pan et al., 2023). Although Aumolertinib has improved the quality of life for patients, there are still individual differences in therapeutic efficacy (Yang et al., 2020; Wu et al., 2021). Especially the adverse reactions caused by the drug, which severely affect the quality of life of patients, including cardiac toxicity, diarrhea, and vision impairment (Jiang et al., 2021; Zhang et al., 2022; Zhou et al., 2023).

Understanding the various factors that influence the pharmacokinetics of a drug is crucial in providing tailored solutions for personalized medical care (van den Anker et al., 2018). Aumolertinib is predominantly metabolized by the cytochrome P450 enzyme (CYP) super family *in vivo*, with CYP3A4 being the primary metabolic enzyme responsible for generating HAS-719, the primary metabolites with lower activity (Liu et al., 2022a; Liu et al., 2022b). It is important to note that the function of CYP3A4 can be altered by genetic factors and drug-drug interactions, thereby affecting the metabolism rate of aumolertinib (Zhou et al., 2005; Tian and Hu, 2014). Therefore, identifying the potential determining factors can promote the rational use of aumolertinib.

At present, there are 48 reported CYP3A4 alleles that have significant frequency differences in their distribution among various ethnic groups (Hu et al., 2017; Guttman et al., 2019). Although *in vitro* enzymatic studies have provided preliminary clarification of the activity of each variant, there is a large gap between basic research and its practical applications, which requires more data to support it (Werk and Cascorbi, 2014). In addition to genetic factors, drug-drug interactions also play an important role in clinical treatment stratification (Malki and Pearson, 2020). These interactions can cause fluctuations in the blood exposure of substrate drugs due to induction or inhibition of CYP3A4 enzyme activity (Boulenc et al., 2016). In this study, we examined the enzymatic kinetics of aumolertinib metabolism based on the novel CYP3A4 variant discovered by our research group previously (Fang et al., 2017). Furthermore, we also screened for commonly used clinical drugs that interact with aumolertinib using a microsomal incubation system, explored the inhibitory mechanism, and discussed the drug interactions in SD rats. Building on this, we overexpressed the wild-type and variant forms of CYP3A4 in the livers of mice to examine the influence of the variant on the metabolism of aumolertinib. The findings of this study not only contribute to our understanding of the enzymatic characteristics of CYP3A4 variants, but also provide insights into their impact on drug interactions.

## 2 Materials and methods

### 2.1 Chemicals and reagents

Aumolertinib (purity>98%) was purchased from Biochempartner (Shanghai, China). HAS-719 (purity>95%) was synthesized by Bioduro-Sundia (Shanghai, China). The schematic diagram of chemical synthesis and identification spectra of HAS-719 are shown in Supplementary Figure S1. Diazepam was manufactured by Shanghai Xudong Haipu Pharmaceutical Co., Ltd (Shanghai, China). Reduced nicotinamide adenine dinucleotide phosphate (NADPH) was purchased from Sigma-Aldrich (St. Louis, Missouri, USA). HPLC-grade acetonitrile and methanol were purchased from Merck (Darmstadt, Germany). Dimethyl sulfoxide (DMSO), and bicinchoninic acid (BCA) protein assay kit was purchased from Beyotime Biotechnology (Shanghai, China). Ultrapure water was purified using a Milli-Q A10 purification system (Billerica, MA, USA). Rat microsomes were extracted according to the preparation method (Wang et al., 2015). Male pooled human liver microsomes, 0.5 mL, were purchased from Corning Life Sciences (#452172, New York, USA).

### 2.2 Conditions for Ultra-high performance liquid chromatography-tandem mass spectrometry (UPLC-MS/MS)

The UPLC-MS/MS system is equipped with a Waters UPLC BEHC18 column (2.1 mm × 50 mm, 1.7 μm particle size). The column and automated sampling rack are maintained at temperatures of 40°C and 4°C, respectively. The mobile phase consists of 0.1% formic acid (A) and acetonitrile (B) and is eluted for 4 min at a flow rate of 0.4 mL/min. The gradient elution is carried out as follows: 90% A (0–0.2 min), 90%–30% A (0.2–1.0 min), 30%–10% A (1.0–2.5 min), 10%–90% A (2.5–2.8 min), and 90% A (2.8–4.0 min). Quantitative analysis is performed using the Waters XEVO TQD triple quadrupole mass spectrometer with Multiple Reaction Monitoring (MRM) detection of analytes in positive mode. The retention time of aumolertinib was 1.85 min, that of HAS-719 was 1.83 min, and that of the internal standard diazepam was 2.34 min (Supplementary Figure S2). The ion pairs monitored for aumolertinib, HAS-719 and diazepam were m/z: 526.01→72.04, m/z: 512.18→455.08, and m/z: 284.91→153.9, respectively (Li et al., 2022; Tang et al., 2022).

### 2.3 Preparation of recombinant human CYP3A4 and CYPb5 cell microsomes

The method for preparing recombinant human CYP3A4 and CYPb5 cell microsomes is the same as we previously reported (Fang et al., 2017; Zhou et al., 2019). Specifically, first the pFastBac-CYPOR-CYP3A4 and CYPb5 plasmids are constructed, then the plasmids are transformed into DH10Bac cells to obtain the bacmid DNA. Subsequently, the bacmid DNA is transfected into Sf21 insect cells to generate high titer recombinant baculoviruses. After infecting the Sf21 cells with the high titer baculovirus, the cell suspension is collected. Finally, the cells are disrupted by sonication, and the microsomes are prepared by ultracentrifugation. To examine the expression of the target proteins and the CYP content, Western blot analysis and carbon monoxide difference spectroscopy were performed, respectively.

### 2.4 Enzymatic incubation assay

The liver microsomal reaction system consists of RLM or HLM, the substrate aumolertinib, an inhibitor, NADPH, and phosphate buffer. When screening for inhibitors, the amount of RLM in the system and the reaction time were optimized and set to 0.5 mg/mL and 30 min, respectively. The concentration of the inhibitor was set at 100 μM. Based on the K_m_ values of the aumolertinib in RLM or HLM (Supplementary Figure S3), the reaction concentration of aumolertinib was set at 25 μM and 20 μM, respectively. The above mixture was pre-incubated at 37 °C for 5 min, and then 1 mM NADPH was added to initiate the reaction, and incubated for another 30 min. Afterwards, 400 μL of cold acetonitrile and 20 μL of diazepam (500 ng/mL) were added to the system to stop the reaction. After vortexing for 2 min, the mixture is centrifuged at 16,200 g for 10 min and the supernatant is subjected to analysis by UPLC-MS/MS for the detection of analytes. To determine the IC_50_, telmisartan and carvedilol are prepared in concentrations of 0, 0.01, 0.1, 1, 10, 25, 50 and 100 μM.

To elucidate whether the inhibition is time-dependent, we conducted an IC_50_ shift experiment. The incubation was divided into two groups. Group A was preincubated for 30 min without substrate and NADPH, while Group B was preincubated for 30 min without substrate. The post-processing method is the same as the one interpreted above.

The reaction system for studying CYP3A4 enzyme kinetics includes 1 pmol CYP3A4 cell microsomes, 10 μg/mL cytochrome b5, aumolertinib (1,2,5,10,20 and 40 μM) and PBS buffer. To determine the IC_50_, telmisartan and carvedilol are prepared at concentrations of 0, 0.01, 0.1, 1, 10, 25, 50 and 100 μM. The subsequent treatment is as above. The reaction start, termination and sample processing are the same as described above.

### 2.5 Investigation of the inhibition mechanism of carvedilol and telmisartan on aumolertinib metabolism

According to the IC_50_ values of the corresponding inhibitors, the concentration of telmisartan was adjusted to 0, 12, 18, and 24 μM for RLM and 0, 25, 75, and 100 μM for HLM. The concentration of carvedilol was adjusted to 0, 28, 42, 56 μM in RLM; 0, 6, 12 and 24 μM in HLM and 0, 0.25, 0.5, and 0.75 μM in CYP3A4. According to the corresponding Michaelis constant, the concentration of aumolertinib is adjusted to 6.25, 12.5, 25 and 50 μM in RLM; 5, 10, 20 and 40 μM in HLM and 0.75,1.5,3 and 6 μM in CYP3A4. The post-processing steps are the same as described in 2.4.

### 2.6 Animal experiment

Male Sprague Dawley (SD) rats (250 ± 15 g) and male C57BL/6J (25.6 ± 1 g) mice were purchased from Vital River Experimental Animal Technology Co., Ltd. (Beijing, China). Considering that female mice have a menstrual cycle, changes in hormone levels may have a significant impact on the results. Since the purpose of this study was not to examine the differences in metabolism between male and female mice, female mice were not included. Before the experiment, all animals were acclimatized for 7 days under standard laboratory conditions with a temperature of 25°C ± 2 °C, relative humidity of 60% ± 5%, and a 12-h light-dark cycle. The animal experiment was approved by the Ethics Committee of Wenzhou Medical University (No. wydw 2023-0351 and wydw 2023-0457).

Rats were fasted for 12 h and had free access to water. Then, they were divided into three groups, n = 6. Two of them orally given carvedilol 5 mg/kg, and telmisartan 8 mg/kg, respectively. Half an hour later, they were administered 11 mg/kg aumolertinib orally, the vehicle for the drugs was corn oil. Tail vein blood samples were collected at 0.5, 1, 2, 3, 4, 6, 8, 12, 24, and 48 h, 200 μL each point. Tail vein blood was centrifuged at 6200 g to obtain the supernatant, which was then stored at −80°C for analysis. 50 μL of plasma, 150 μL of acetonitrile and 20 μL of diazepam (500 ng/mL) were mixed, vortexed for 2 min, and centrifuged at 16,200 g for 10 min.

63 C57BL/6J mice were randomly divided into three groups (21 mice each group), and AAV8-Control, AAV8-CYP3A4*1 virus and AAV8-CYP3A4*12 virus were injected into the tail vein, respectively. The virus amount per mouse is 2.5×10^11^ VG, and the injection volume is 200 μL. Animal experiments were carried out after 4 weeks, each group is further divided into three subgroups (n = 7). Before the experiment, the mice fasted for 12 h and drank copiously. They then received 7.5 mg/kg carvedilol or 12 mg/kg telmisartan, and half an hour later, aumolertinib (16.5 mg/kg) was administered orally. After 0.5, 1, 2, 4, 8, 24, 36, and 48 h, blood was collected from the fundic vein, then centrifuged at 2400 g for 10 min, and the supernatant was stored at −80°C. To measure serum concentration, 10 μL of serum was taken, 30 μL of ACN and 20 μL of diazepam (500 ng/mL) were added, the mixture was vortexed for 2 min, then centrifuged at 16,200 g for 10 min, and 40 μL of the supernatant was mixed with 40 μL of pure water, and the resulting liquid was then injected into the instrument for analysis. For subsequent frozen sectioning and imaging, the mouse liver was removed and placed in 4% paraformaldehyde. Finally, the slice was stained with DAPI, and observed using a fluorescence microscope.

### 2.7 Statistical analysis

The Michaelis-Menten equation curve was created using GraphPad Prism 9.0 software. Drug and Statistics (DAS) software (version 3.0, Bontz Inc., Beijing, China) was used for statistical moment parameter analysis to a noncompartmental model to obtain the pharmacokinetic properties. All data were presented as mean ± standard deviation. In the one-way ANOVA analysis, Dunnett’s t-test was used for statistical comparison of the differences between CYP3A4 variants. The unpaired t-test was used to compare the *in vivo* pharmacokinetic parameters between different groups using SPSS 26.0. A *p* ≤ 0.05 indicated a statistically significant difference.

## 3 Results

### 3.1 Clarify the drug interaction spectrum of aumolertinib

**FIGURE 1:**
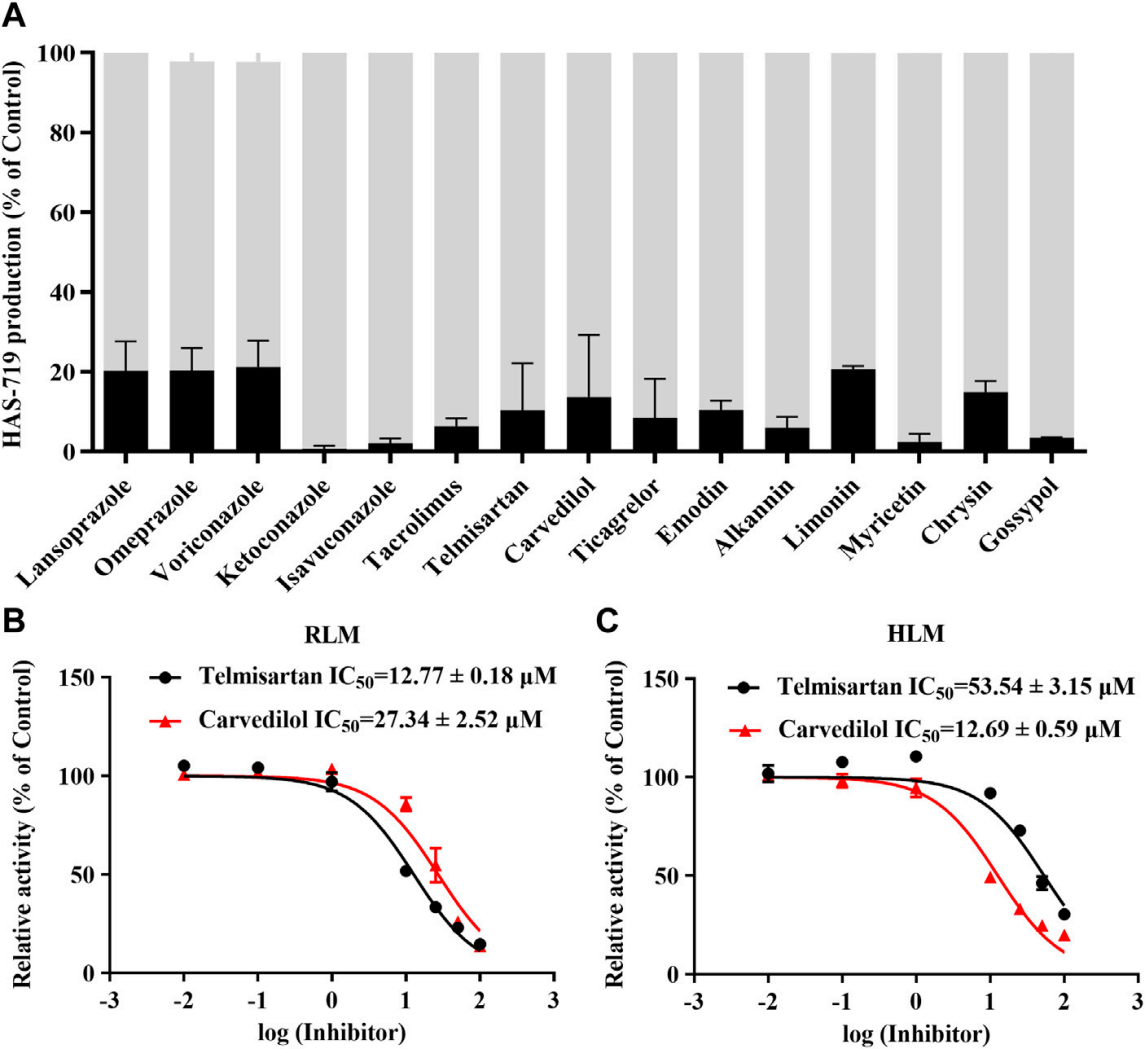
The drug spectrum that inhibits the metabolism of aumolertinib. **(A)** RLM was used to screen the drug interactions with Aumolertinib. The drugs with the inhibition rate ≥80% are shown. Efficacy of telmisartan, carvedilol on inhibiting aumolertinib in different enzymatic system. **(B, C)** The IC_50_ values of telmisartan and carvedilol were determined using RLM, HLM, Data are presented as mean ± SD, n = 3.

To further clarify the drugs that may interact with aumolertinib, we examined 153 clinically used drugs and natural products (Supplementary Table S1). As shown in Figure 1A, we identified 15 drugs that could significantly inhibit the metabolism of aumolertinib (% of control ≤20%), including the classic CYP enzyme inhibitors ketoconazole, isavuconazole, and omeprazole. Interestingly, we also noted that two classic cardiovascular drugs, telmisartan and carvedilol, were among these inhibitors. Considering the high incidence and poor prognosis of cardiovascular diseases in cancer patients, investigating the interactions between antitumor drugs and cardiovascular drugs is clinically relevant. We subsequently examined the inhibitory effects of telmisartan and carvedilol on the metabolism of aumolertinib. In RLM, the IC_50_ of telmisartan and carvedilol were 12.77 ± 0.18 μM and 27.34 ± 2.52 μM, respectively (Figure 1B). In HLM, they were 53.54 ± 3.15 μM and 12.69 ± 0.59 μM, respectively, as shown in Figure 1C.

### 3.2 Telmisartan and carvedilol significantly increase the blood exposure of aumolertinib

**FIGURE 2:**
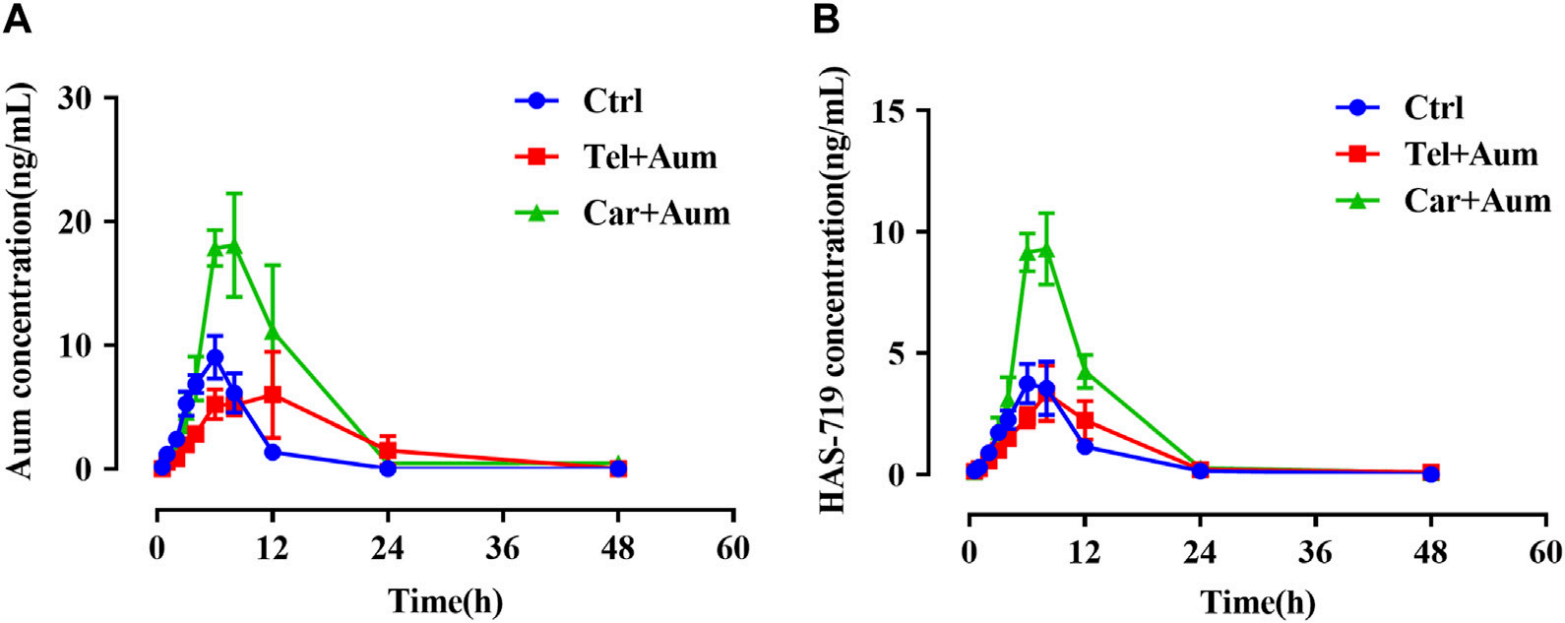
Mean concentration-time curves of aumolertinib and HAS-719. Pharmacokinetics study was performed using SD rats as indicated in the Methods section. Time–concentration curves of aumolertinib **(A)** and metabolite HAS-719 **(B)** were plotted. Data are presented as mean ± SEM, n = 6. *p < 0.05, **p < 0.01, ***p < 0.001.

We then further examined the effects of telmisartan and carvedilol on the metabolism of aumolertinib *in vivo*. As Figure 2A showed, the pharmacokinetic profile of the combined group significant different from the aumolertinib alone group. The AUC_(0-t)_ of aumolertinib increased more than 3-time, while V_z/F_ and CL_z/F_ decreased and C_max_ increased after combination with carvedilol (Table 1). In addition, telmisartan significantly prolonged the peak time of aumolertinib. Likewise, the pharmacokinetic curve of HAS-719 showed no significant changes in the telmisartan combination group, while the blood concentration of HAS-719 increased almost 2-time in the carvedilol combination group, as shown in Figure 2B. Similarly, in terms of metabolic rate, after combined use with telmisartan and carvedilol, both AUC ratio and T_max_ decreased remarkable (Table 1).

**TABLE 1:** The main pharmacokinetic parameters and their ratio (metabolite/parent ratio) of Aumolertinib and HAS-719 in three groups of SD rats.

Group	Aumolertinib			HAS-719		
Parameters	Aum	Tel + Aum	Car + Aum	Aum	Tel + Aum	Car + Aum
AUC_(0-t)_ (μg/L·h)	65.89 ± 27.62	153.17 ± 175.31	198.07 ± 121.40*	38.40 ± 17.84	41.38 ± 26.78	92.86 ± 22.46***
AUC_(0-∞)_ (μg/L·h)	65.89 ± 27.63	153.18 ± 175.31	203.21 ± 133.07	38.41 ± 17.84	44.83 ± 29.18	93.32 ± 22.55***
t_1/2z_ (h)	2.62 ± 0.94	3.21 ± 0.24	4.44 ± 2.79	3.69 ± 0.57	6.60 ± 7.58	4.25 ± 1.98
T_max_ (h)	5.17 ± 1.33	8.67 ± 2.73*	7.33 ± 2.42	6.33 ± 0.82	8.33 ± 1.97*	7.00 ± 1.10
V_z/F_ (L/kg)	675.07 ± 272.65	582.21 ± 298.65	374.52 ± 139.60*	1,913.68 ± 1,058.91	2,414.43 ± 1,605.18	763.01 ± 377.55*
CL_z/F_ (L/h/kg)	185.62 ± 56.47	123.66 ± 63.04	68.94 ± 29.85**	345.03 ± 157.05	339.77 ± 197.93	123.40 ± 27.94*
C_max_ (μg/L)	9.48 ± 3.93	8.73 ± 7.55	21.81 ± 8.00*	4.30 ± 2.66	3.59 ± 2.70	10.53 ± 2.96**

metabolite/parent ratio			
Group	Aum	Tel+Aum	Car+Aum
Parameters			
AUC_(0–t)_ (μg/L·h)	0.58±0.27	0.27±0.17*	0.47±0.11
AUC_(0–∞)_ (μg/L·h)	0.58±0.27	0.29±0.19	0.46±0.11
t_1/2z_ (h)	1.40±0.22	2.06±2.36	0.96±0.45
T_max_ (h)	1.23±0.16	0.96±0.23*	0.95±0.15*
V_z/F_ (L/kg)	2.83±1.57	4.15±2.76	2.04±1.01
CL_z/F_ (L/h/kg)	1.86±0.85	2.75±1.60	1.79±0.41
C_max_ (μg/L)	0.45±0.28	0.41±0.31	0.48±0.14

Metabolite/parent ratio.

### 3.3 Telmisartan and carvedilol inhibit the metabolism of aumolertinib through a mixed mechanism

**FIGURE 3:**
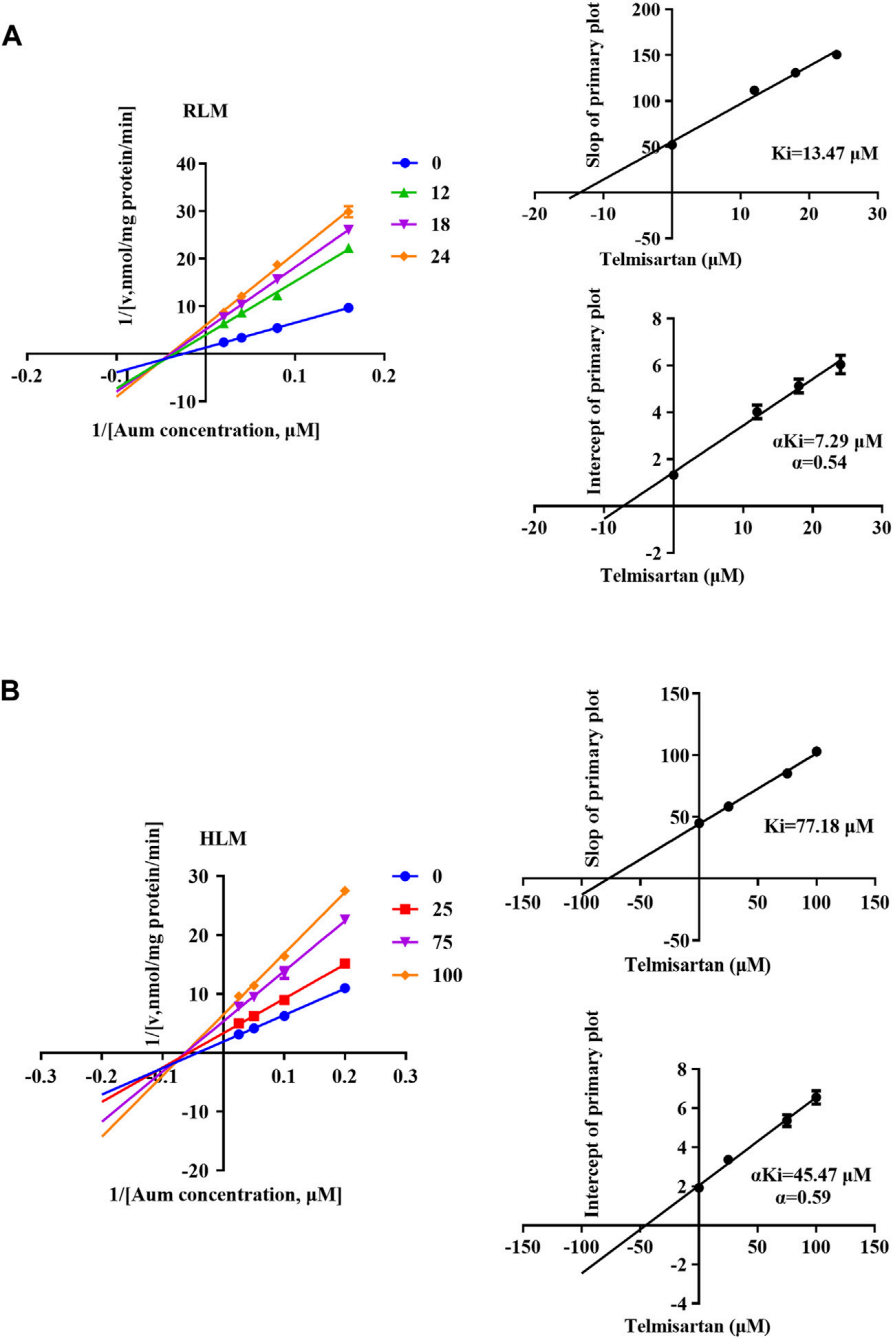
Lineweaver–Burk plot, the secondary plot for Ki, and the secondary plot for αKi for the inhibition of Aumolertinib metabolism by telmisartan, with various concentrations in rat liver microsome **(A)** and in human liver microsome **(B)**. Data are presented as mean ± SD, n = 3.

**FIGURE 4:**
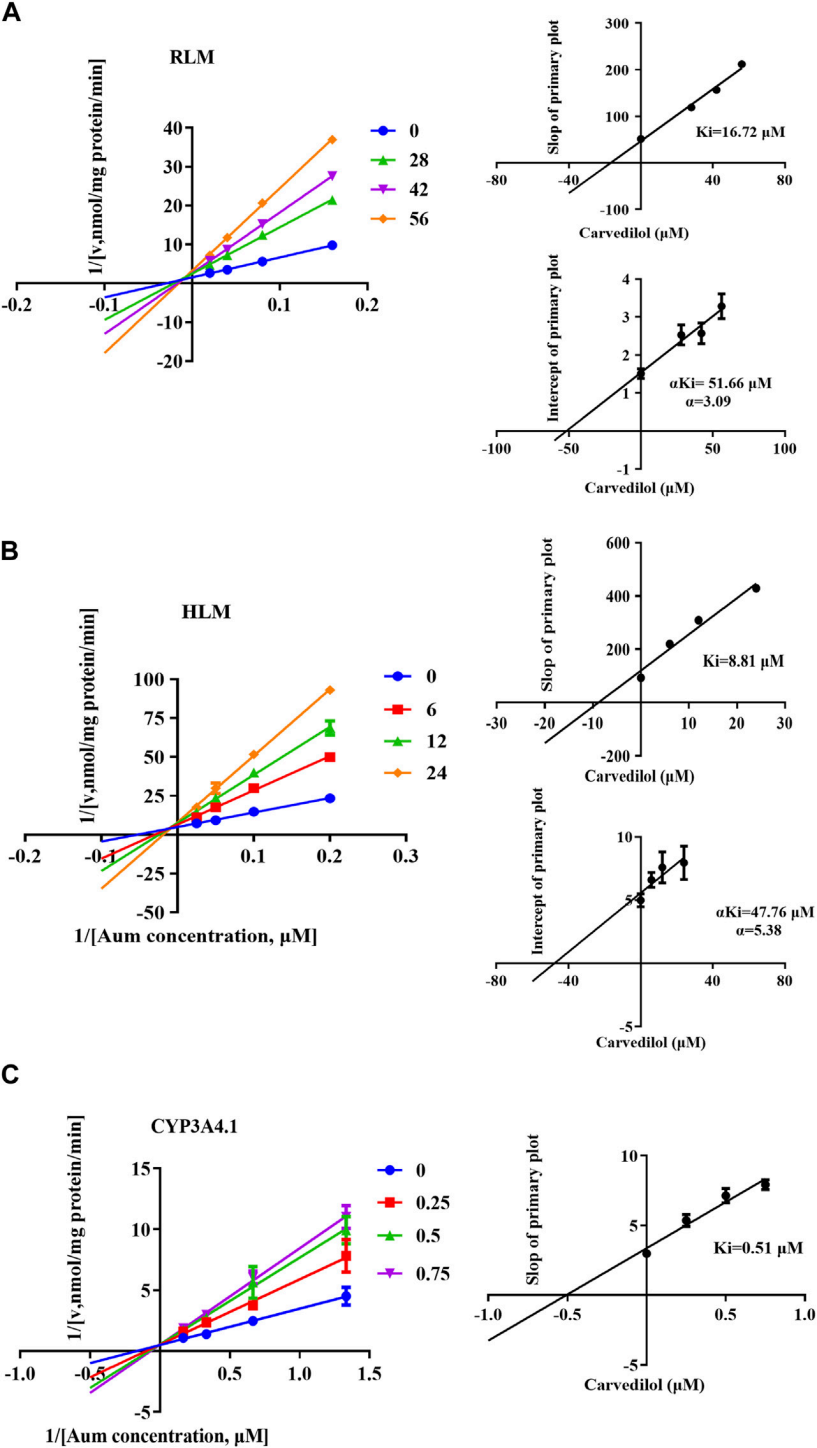
Lineweaver–Burk plot, the secondary plot for Ki, and the secondary plot for αKi for the inhibition of Aumolertinib metabolism by carvedilol, with various concentrations in rat liver microsome **(A)** and in human liver microsome **(B)**, with various concentrations in CYP3A4.1 **(C)**. Data are presented as mean ± SD, n = 3.

To clarify the mechanism of interaction, we first performed IC_50_ shift experiments to determine whether the inhibition was time dependent (Xu et al., 2023). The results showed that the ratio of IC_50_ (-NADPH) to IC_50_ (+NADPH) of telmisartan and carvedilol in RLM was less than 1.5, indicating that the inhibition was not time dependent (Supplementary Figure S4). We then performed enzymatic kinetics studies and performed double reciprocal processing of the Michaelis-Menten equation. As shown in Figures 3A,B, telmisartan exhibited mixed (non-competitive and uncompetitive) inhibition mechanisms with Ki values of 13.47 and 77.17 μM in RLM and HLM, respectively, with α < 1. Carvedilol exhibited non-competitive and competitive mixed inhibition mechanisms with Ki values of 16.72 and 8.81 μM in RLM and HLM, respectively, with α > 1, as shown in Figures 4A,B. In addition, we examined the inhibitory effect of carvedilol on human recombinant CYP3A4.1, and the results indicated that it was competitively inhibited (Figure 4C).

### 3.4 The genetic polymorphism of CYP3A4 determines the enzyme kinetic parameters of aumolertinib

**FIGURE 5:**
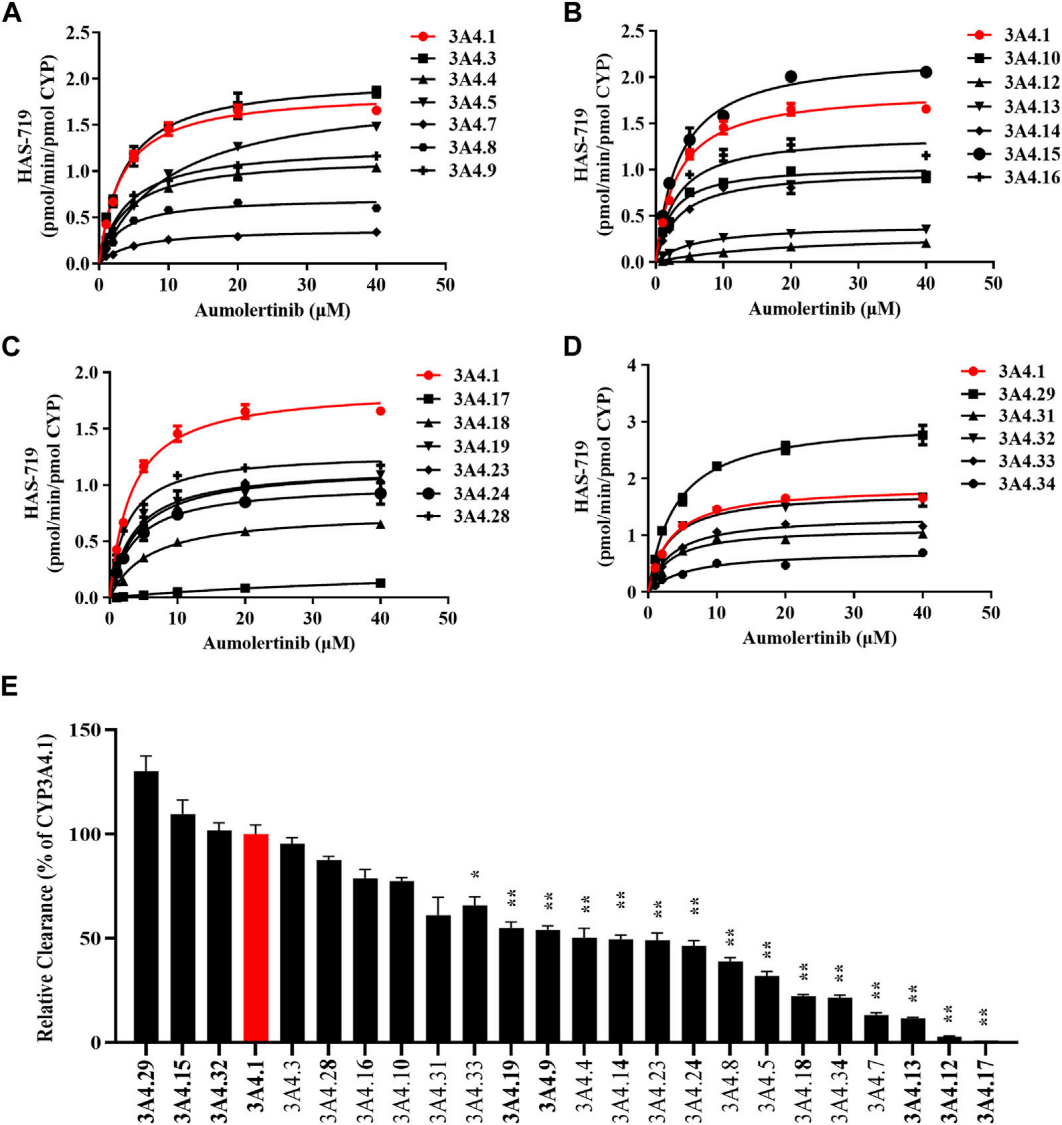
Kinetics profile of CYP3A4 variant on catalyzing aumolertinib. **(A–D)** The microsomal enzymatic incubation assay was performed as indicated in the methods section. The Michaelis–Menten curves were plotted. **(E)** Relative clearance of CYP3A4 was determined based on the kinetics parameters. Data are presented as mean ± SD, n = 3. *p < 0.05, **p < 0.01, ***p < 0.001.

To clarify the enzyme kinetic properties of aumolertinib metabolism, we generated 24 CYP3A4 variants using a baculovirus-insect cell expression system and then used an *in vitro* enzyme incubation method to determine the enzyme kinetic parameters. Figures 5A–D show the Michaelis-Menten curves of each variant in the metabolism of aumolertinib. In CYP3A4.1, V_max_, K_m_, and Cl_int_ were 1.8 pmol/min/pmol, 3.2 μM and 0.58 μL/min/pmol CYP, respectively. Among them, the relative clearance rates of CYP3A4.29 and CYP3A4.15 were higher than those of wild type, while CYP3A4.3, 10, 16, 28, 31 and 32 were not significantly different from those of wild type. In contrast, the relative clearance rates of the remaining variants, including CYP3A4. 4, 5, 7, 8, 9, 12, 13, 14, 17, 18, 19, 23, 24, 33, and 34 were significantly decreased, as shown in Figure 5E. Compared to the control group, the changes in V_max_ of the variants ranged from 17.0% to 163.6%, and the relative percentage changes in K_m_ ranged from 100.2% to 181.9%. Only the K_m_ values of 8, 10, 16, 28, and 32 were smaller than those of the wild type, ranging from 72.2% to 98.6%, as shown in Table 2.

**TABLE 2:** Kinetic parameters of aumolertinib catalyzing by CYP3A4.

Variants	Vmax (pmol/min/pmol P450)	Km (μM)	CLint (Vmax/Km) (μl/min/pmol P450)
*1	1.866 ± 0.036	3.204 ± 0.202	0.583 ± 0.026
*3	2.020 ± 0.032	3.631 ± 0.149	0.557 ± 0.017
*4	1.153 ± 0.018**	3.952 ± 0.389	0.293 ± 0.027**
*5	1.882 ± 0.038	10.144 ± 0.833*	0.186 ± 0.012**
*7	0.377 ± 0.016***	4.937 ± 0.649	0.077 ± 0.006**
*8	0.717 ± 0.016***	3.158 ± 0.077	0.227 ± 0.010**
*9	1.280 ± 0.031**	4.080 ± 0.370	0.315 ± 0.023**
*10	1.045 ± 0.010**	2.314 ± 0.065	0.452 ± 0.009
*12	0.318 ± 0.031***	19.773 ± 4.091	0.016 ± 0.002**
*13	0.405 ± 0.007**	6.015 ± 0.384*	0.067 ± 0.003**
*14	0.996 ± 0.063**	3.445 ± 0.109	0.289 ± 0.012**
*15	2.260 ± 0.024**	3.540 ± 0.181	0.640 ± 0.039
*16	1.382 ± 0.024**	3.013 ± 0.205	0.460 ± 0.024
*17	0.321 ± 0.058***	58.283 ± 13.120	0.006 ± 0.000**
*18	0.759 ± 0.008**	5.859 ± 0.286**	0.130 ± 0.005**
*19	1.160 ± 0.015**	3.625 ± 0.237	0.321 ± 0.017**
*20	N.D	N.D	N.D
*23	1.163 ± 0.025***	4.085 ± 0.391	0.286 ± 0.021**
*24	1.012 ± 0.074**	3.732 ± 0.252	0.271 ± 0.014**
*28 (*L22V)	1.287 ± 0.005*	2.519 ± 0.054	0.511 ± 0.010
*29 (*F113I)	3.053 ± 0.121*	4.031 ± 0.319	0.759 ± 0.043
*31 (*H324Q)	1.129 ± 0.016**	3.213 ± 0.502	0.356 ± 0.050
*32 (*I335T)	1.759 ± 0.066	2.963 ± 0.092	0.594 ± 0.021
*33 (*A370S)	1.345 ± 0.079*	3.520 ± 0.403	0.384 ± 0.024*
*34 (*I427V)	0.730 ± 0.028***	5.817 ± 0.439	0.126 ± 0.007**

Note. compared to wild type, *p < 0.05; **p < 0.01; ***p < 0.001. ND, not determined.

### 3.5 The activity of CYP3A4 also determines the inhibitory potency of telmisartan and carvedilol on the metabolism of aumolertinib

**FIGURE 6:**
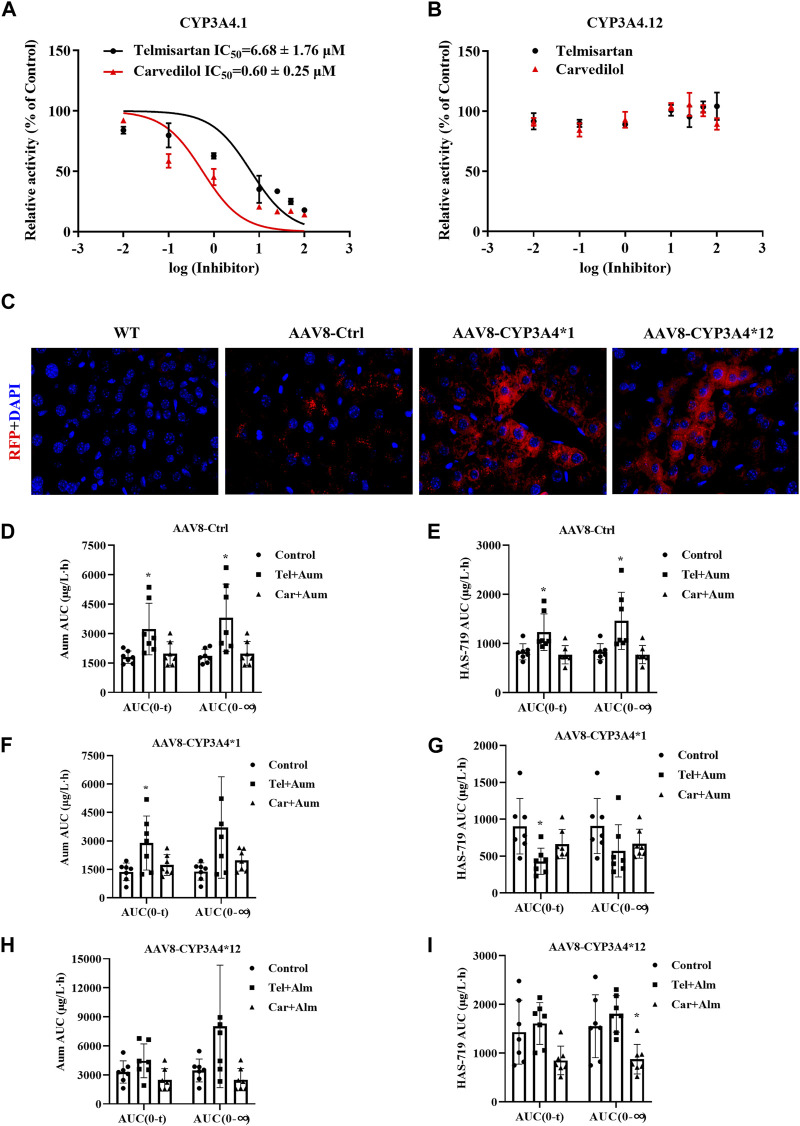
The interaction between Telmisartan, Carvedilol, and aumolertinib in liver-specific CYP3A4 overexpressing mice. **(A, B)** The IC_50_ values of telmisartan and carvedilol were determined using CYP3A4.1. n = 3. **(C)** The mice liver frozen section, magnification ×40. The histogram of AUC pharmacokinetic parameters of aumolertinib in each group, AAV8-Ctrl **(D, E)**; AAV8-CYP3A4.1 **(F, G)**; AAV8-CYP3A4.12 **(H,I)**. Data are presented as mean ± SD, n = 7. *p < 0.05, **p < 0.01, ***p < 0.001.

The different CYP3A4 variant enzymes exhibit markedly different catalytic activities towards the metabolism of aumolertinib. However, it remains unclear whether these CYP3A4 variants also influence the drug-drug interactions involving aumolertinib. Therefore, we subsequently selected CYP3A4*1 and CYP3A4*12 (which is more prevalent in Asian populations) to further investigate this aspect. As shown in Figures 6A,B, in CYP3A4.1 the IC_50_ values of telmisartan and carvedilol decrease to 6.68 ± 1.76 μM and 0.60 ± 0.25 μM, respectively, while in CYP3A4.12, the IC_50_ values were above 100 μM.

To further investigate the influence of different CYP3A4 variants on drug interactions, we constructed liver-specific adenoviruses and successfully obtained AAV8-CYP3A4*1 and AAV8-CYP3A4*12 mice. As shown in Figure 6C, in the AAV8-CYP3A4*1 and AAV8-CYP3A4*12 mice, the expression levels of the target proteins were comparable. We then performed pharmacokinetic studies. The pharmacokinetic curves of the substrates and the metabolite showed significant differences between mice with different genotypes. In mice injected with blank viruses, co-administration of carvedilol resulted in varying degrees of decrease in t_1/2_, V_z/F_ and C_max_ of aumolertinib, while T_max_ increased significantly. For its metabolite HAS-719, T_max_ increased significantly while C_max_ decreased, as shown in Figures 6D,E and Supplementary Figure S5A. Compared to the control group, mice co-administered with telmisartan and aumolertinib showed a doubling of both AUC and T_max_. The pharmacokinetic curves showed a clear bimodal distribution in the group receiving carvedilol and telmisartan simultaneously. Based on this, it is less meaningful to discuss the change of t_1/2_, and we emphasize the change of AUC. In mice with wild-type CYP3A4 overexpression, the AUC of aumolertinib increase significantly when co-administered with telmisartan, T_max_ increased. The AUC and C_max_ of HAS-719 decreased, indicating that telmisartan inhibited the metabolism of aumolertinib in mice with wild-type CYP3A4 overexpression, as shown in Figures 6F,G and Supplementary Figure S5B. In mice with CYP3A4*12 overexpression, except for a slight decrease in t_1/2_ in the carvedilol group, there were no significant differences in the pharmacokinetics of aumolertinib among the co-administration groups, suggesting no or weak drug interactions, as shown in Figures 6H,I and Supplementary Figure S5C. The specific pharmacokinetic parameters of mice are in the table 2-4 in the supplementary document.

In mice injected with blank viruses, the metabolic rate has not changed obviously, except for C_max_ after combined application with carvedilol (Supplementary Table S2). In mice with wild-type CYP3A4 overexpression, after combined use of telmisartan, AUC ratio decreased significantly, while CL_z/F_ was the opposite. After combined application of carvedilol, V_z/F_ and CL_z/F_ increased (Supplementary Table S3). In mice with CYP3A4*12 overexpression, the ratio of metabolic rate is basically unchanged (Supplementary Table S4).

## 4 Discussion

Aumolertinib is a third-generation EGFR inhibitor that has demonstrated good efficacy against EGFR inhibitor-resistant mutations, including T790M and L858R (Yang et al., 2020; Wu C. P. et al., 2021; Ge et al., 2021; Huang and Wang, 2022; Zhang et al., 2023). Studies have shown that aumolertinib is primarily metabolized by the liver, producing metabolites such as M4, M5, M6, and M7. Among them, M5 (HAS-719) is the main active metabolite, with a plasma exposure of 40% of the parent drug (Zhou et al., 2021; Liu et al., 2022a; Liu et al., 2022b). It has been reported that CYP3A4 is the main enzyme system responsible for catalyzing the generation of HAS-719 from aumolertinib. Due to its rich genetic polymorphism and susceptibility to drug induction or inhibition, changes in CYP3A4 enzyme activity can lead to fluctuations in aumolertinib blood exposure and affect efficacy (Zhou, 2008; Fu et al., 2023). Although other factors, such as food, gender, age, and more, can also influence the pharmacokinetic characteristics of drug metabolism, these aspects were not the focus or within the scope of the present study (Hoover et al., 2016).

Through screening of a drug library, we have identified several drugs that can significantly inhibit the metabolism of aumolertinib, including proton pump inhibitors, azole antifungals, and cardiovascular drugs. Among them, drugs like ketoconazole and isavuconazole exhibited very potent inhibitory effects. Their primary mechanism of action is to inhibit fungal CYP enzyme activities, which are well-recognized as liver enzyme activity inhibitors (Bellmann and Smuszkiewicz, 2017). Therefore, they can also be used as positive controls, demonstrating the reliability of the *in vitro* enzymatic reaction system. It is worth noting that concurrent fungal infections are quite common in cancer patients (Groll et al., 2021). In such cases, it is recommended to appropriately reduce the dose of aumolertinib. Gastrointestinal adverse reactions are also common during cancer treatment, and the co-administration of proton pump inhibitors is a common strategy. Our previous research has confirmed that omeprazole and other proton pump inhibitors can inhibit the metabolism of osimertinib both *in vitro* and *in vivo* (Gao et al., 2022). Considering all these factors, we ultimately focused our research on carvedilol and telmisartan.

Based on the results, carvedilol and telmisartan can both inhibit the metabolism of aumolertinib, but the inhibition potency differs between RLM (rat liver microsomes) and HLM (human liver microsomes). Carvedilol exhibited stronger inhibition against HLM, while telmisartan showed the opposite. In terms of the inhibition mechanism, both compounds exhibited mixed-type inhibition in both RLM and HLM. However, there were some differences. Carvedilol was found to be a competitive and noncompetitive mixed-type inhibitor. Therefore, we further investigated its inhibitory effect on CYP3A4.1, and the results showed that this inhibition was competitive in nature. The different inhibition mechanisms observed between the two reaction systems (RLM and HLM) suggest that the metabolism of aumolertinib may involve pathways other than just the CYP3A4 pathway. In other words, aumolertinib may have multiple metabolic routes, and the inhibition by carvedilol and telmisartan may not be solely dependent on the CYP3A4 enzyme. The pharmacokinetics of aumolertinib in rats suggest that the effects of telmisartan and carvedilol are different. Specifically, carvedilol not only increases the plasma concentration of aumolertinib, but also increases the systemic exposure of its metabolites. This is likely due to its inhibition of intestinal CYP3A4, which leads to increased drug absorption and improved bioavailability. From the data on the drug metabolic ratios in the blood, the effects of telmisartan and carvedilol are consistent with their expected inhibitory effects. From a clinical perspective, the probability of cancer patients having concurrent cardiovascular diseases is quite high, and their prognosis is often poorer (Willems et al., 2022). Therefore, the combination of aumolertinib with carvedilol or telmisartan is a clinically relevant possibility. This project has provided fundamental research data on the potential drug-drug interactions and early warnings regarding the concomitant use of aumolertinib with these cardiovascular drugs. This information can help guide clinicians in making more informed decisions and managing potential adverse effects when prescribing these combinations for cancer patients with comorbid cardiovascular conditions. In summary, the findings from this study lay the groundwork for further clinical investigations and considerations regarding the safe and effective use of aumolertinib in complex cancer patients with multiple comorbidities.

In addition, we examined the enzyme kinetics parameters of 24 CYP3A4 variants in metabolizing aumolertinib and found that the enzyme kinetics parameters of CYP3A4.4, 5, 7, 8, 9, 12, 13, 14, 17, 18, 19, 23, 24, 33, and 34 changed significantly, and the relative clearance rate was significantly reduced, especially CYP3A4.12 and 17 almost lost function. These results suggest that people carrying the above CYP3A4 allele genes need to appropriately reduce the dosage of aumolertinib. There are notable discrepancies in the distribution of CYP3A4 alleles among various ethnic groups. In Asian populations, the CYP3A4*1 allele is the most prevalent, with a frequency of approximately 0.5. Meanwhile, the frequencies of CYP3A4*4, CYP3A4*5, CYP3A4*12, CYP3A4*17, and CYP3A4*18 are relatively high, ranging from 0.05 to 0.15 (Hu et al., 2006; Lee et al., 2007; Zhou et al., 2011; McGraw and Waller, 2012; Saiz-Rodríguez et al., 2020; Fohner et al., 2021). Similarly, CYP3A4*1 is the most frequent allele in Europeans, with a higher frequency of around 0.7, Additionally, CYP3A4*4, CYP3A4*5, CYP3A4*19, and CYP3A4*23 have higher frequencies, ranging from 0.05 to 0.1 (García-Martín et al., 2002; Zhou and Lauschke, 2022). In African populations, CYP3A4*1 relatively common (Chowbay et al., 2005; Drögemöller et al., 2013). It is crucial to note that the corresponding variant’s activity for metabolizing aumolertinib is generally decreased, emphasizing the significance of assessing a patient’s CYP3A4 genotype to facilitate personalized medication.

According to the metabolic difference of aumolertinib among CYP3A4 variants, the relative clearance rates of CYP3A4.12 and CYP3A4.17 were significantly reduced, and the enzyme activity was poor. Considering that CYP3A4 has been reported as a defective allele protein due to the decrease of the activity of the first CYP3A4 substrate by more than 99% (Lee et al., 2005; Lee and Goldstein, 2005), we chose the type 12 with the lowest relative activity except CYP3A4 for subsequent experiments. In the CYP3A4 wild type, the IC_50_ values of telmisartan, carvedilol for inhibiting the metabolism of aumolertinib were in the low range, while in the variants CYP3A4.12, this inhibitory effect was not observed significant. According to the metabolic data of wild-type and mutant mice overexpressing CYP3A4, the metabolism of aumolertinib was influenced by the interaction of carvedilol and telmisartan in different degrees in both wild-type and blank-virus-injected mice, but in the overexpression CYP3A4*12 type, the group using carvedilol and telmisartan in combination did not show inhibitory effect on the metabolism of aumolertinib, suggesting that it had low inhibitory ability in the mutant with low activity. The results suggest that the polymorphism of CYP3A4 can change the potency of drug-drug interaction.

## 5 Conclusion

Carvedilol and telmisartan can significantly inhibit the metabolism of aumolertinib, thereby increasing its systemic exposure. This effect is related to their inhibition of hepatic drug-metabolizing enzymes, particularly CYP3A4. Furthermore, the genetic polymorphisms of CYP3A4 can also significantly alter the pharmacokinetic parameters of aumolertinib, and influence the extent to which carvedilol and telmisartan inhibit the metabolism of aumolertinib. Based on the pharmacokinetic characteristics revealed in this study, the findings provide fundamental data to support the precise application of aumolertinib, especially in the context of potential drug-drug interactions and the impact of CYP3A4 genetic variability.

## Data Availability

The original contributions presented in the study are included in the article/Supplementary Material, further inquiries can be directed to the corresponding authors.

## Abbreviations

AUC, area under the blood concentration–time curve; CL_z/F_, blood clearance; C_max_, maximum blood concentration; CYP450, cytochrome P450; DDIs, drug–drug interactions; DAS, drug and statistics; RLM, rat liver microsomes; HLM, human liver microsomes; IC_50_, half-maximal inhibitory concentration; Ki, inhibition constant; Km, Michaelis–Menten constant; CL_int_, intrinsic clearance, nicotinamide adenine dinucleotide phosphate; SPSS, Statistical Package for Social Sciences; T_max_, peak time; t_1/2z_, elimination half time; UPLC-MS/MS, ultra-performance liquid chromatography–tandem mass spectrometry; V_max_, maximum velocity of the reaction; V_z/F_, apparent volume of distribution; PBS, Phosphate Buffered Saline; ACN, Acetonitrile; SD, Sprague-Dawley; PMSF, Phenylmethanesulfonyl fluoride.
