# Supplementary material for: The influence of drug-induced metabolic enzyme activity inhibition and CYP3A4 gene polymorphism on aumolertinib metabolism
Source: Front Pharmacol. 2024 May 24;15:1392849. doi: 10.3389/fphar.2024.1392849 (PMC11157048; doi:10.3389/fphar.2024.1392849)
Supplement: Supplementary file 1 [file DataSheet1.docx]

***Supplementary information***

A

B

**The influence of drug-induced metabolic enzyme activity inhibition and CYP3A4 gene polymorphism on aumolertinib metabolism**

Supplementary Figures: 5

Supplementary table: 4

A


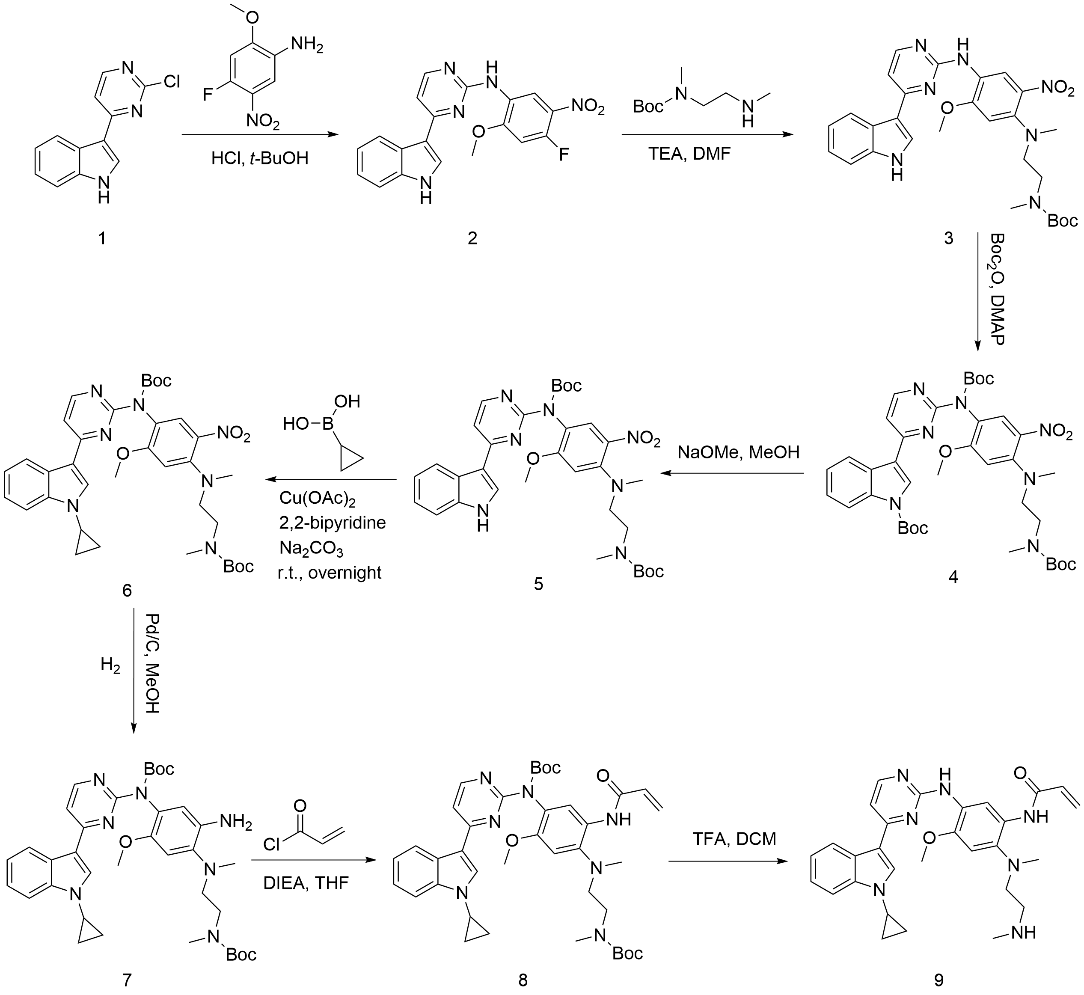


B

Bruker Avance III: 400 MHz

Solvent:DMSO-*d*6

C


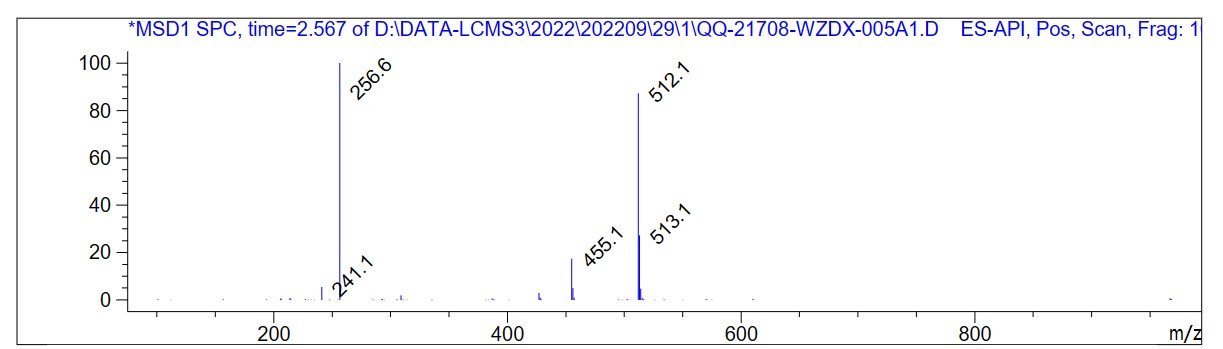


**Supplementary Figure 1.** Chemical synthesis schematic of HAS-719 and nuclear magnetic resonance and mass spectrometry of HAS-719

Aumolertinib

HAS-719

B

A


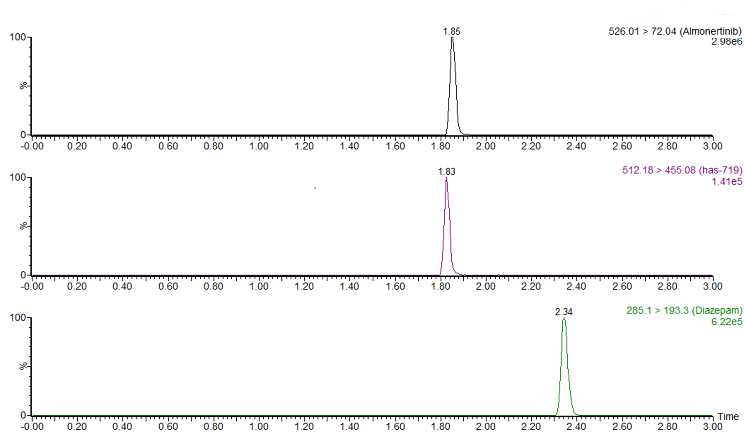

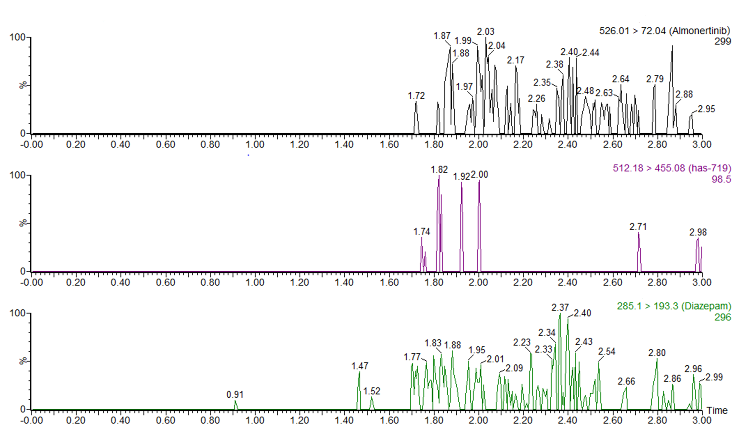


Aumolertinib

Aumolertinib

HAS-719

HAS-719

Diazepam

Diazepam

C


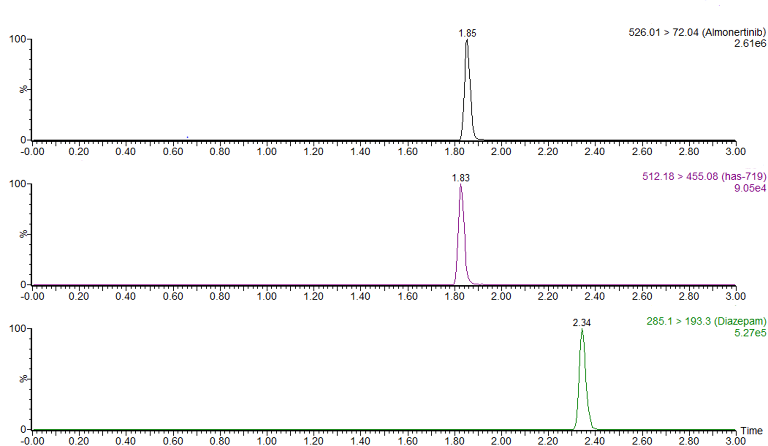


Aumolertinib

HAS-719

Diazepam

**Supplementary Figure 2**. Metabolic Pathway of aumolertinib and the representative chromatogram of analytes. B Chromatogram of a plasma blank sample. C Chromatogram of a plasma blank sample spiked with aumolertinib and HAS-719. D Chromatogram of a plasma sample after the administration of aumolertinib.


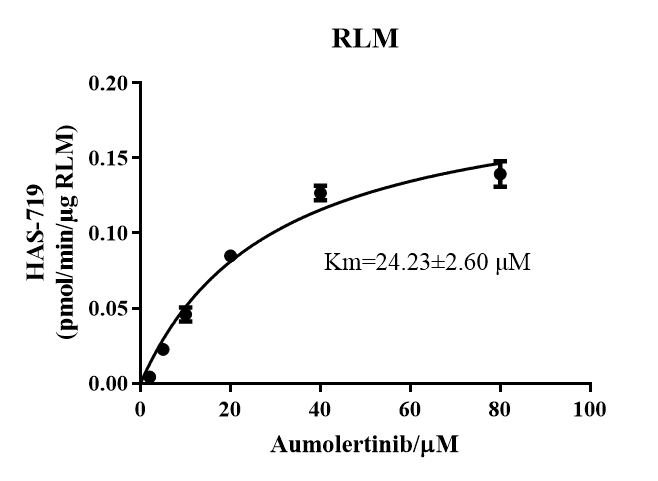


B

A

**Supplementary Figure 3.** The Michaelis–Menten curve for Aumolertinib in rat liver microsome (RLM) and human liver microsome (HLM). Data are presented as mean ± SD, n = 3.

A

B

**Supplementary Figure 4.** The IC_50_ shift curves of carvedilol (A) and telmisartan (B) on rat liver microsome (RLM) activity.


A

B

C

**Supplementary Figure 5**. The interaction between Telmisartan, Carvedilol, and aumolertinib in liver-specific CYP3A4 overexpressing mice. Mean concentration-time curves of aumolertinib and HAS-719. Pharmacokinetics study was performed using mice as indicated in the Methods section. Time–concentration curves of aumolertinib were plotted. Data are presented as mean ± SEM, n = 7. **P* < 0.05, ** *P* < 0.01, *** *P* < 0.001.

**Supplementary Table 1.** Information of screened drug

| Drug name | Molecular weight | of control(%) |
| --- | --- | --- |
| Lansoprazole | 369.36 | 20.29% |
| Paliperidone | 426.48 | 55.22% |
| Loperamide Hydrochloride | 513.50 | 30.23% |
| Omeprazole | 345.42 | 20.33% |
| Cimetidine | 252.34 | 81.40% |
| Rabeprazole | 359.44 | 27.33% |
| Fluvoxamine | 318.33 | 91.86% |
| Voriconazole | 349.31 | 21.23% |
| Ketoconazole | 530.15 | 0.67% |
| Isavuconazole | 437.47 | 2.12% |
| Fluconazole | 306.27 | 40.12% |
| Posaconazole | 700.78 | 27.33% |
| Itraconazole | 705.64 | 103.49% |
| Azithromycin | 748.99 | 103.49% |
| Tacrolimus | 804.03 | 6.38% |
| Erythromycin | 733.93 | 102.33% |
| Clarithromycin | 747.96 | 138.95% |
| Ciprofloxacin Hydrochloride | 385.82 | 94.41% |
| Furazolidone | 225.16 | 98.50% |
| Ofloxacin | 361.37 | 93.80% |
| Cefradine | 349.40 | 101.74% |
| Sitafloxacin | 409.81 | 95.96% |
| Paracetamol | 151.16 | 113.14% |
| Valdecoxib | 314.36 | 43.82% |
| Isopropiram Fumarate | 391.45 | 87.28% |
| Parecoxib | 370.42 | 73.31% |
| Nefopam Hydrochloride | 289.80 | 77.12% |
| Diclofenacsodium | 318.13 | 80.36% |
| Celebrex | 381.37 | 25.62% |
| Chlorzoxazone | 169.57 | 74.71% |
| Cetacort | 362.46 | 72.87% |
| Lornoxicam | 371.82 | 75.72% |
| Lacosamide | 250.29 | 101.63% |
| Meloxicam | 351.42 | 87.72% |
| Tapentadol Hydrochloride | 257.80 | 105.47% |
| Flurbiprofen | 244.26 | 96.76% |
| Indomethacin | 357.79 | 80.94% |
| loxoprofen | 246.31 | 93.55% |
| Dexamethasone | 392.47 | 84.97% |
| Agomelatine | 243.31 | 75.41% |
| Amitriptyline | 313.86 | 98.91% |
| Bupropion hydrochloride | 276.20 | 99.66% |
| Venlafaxine Hydrochloride | 313.86 | 96.08% |
| Brexpiprazole | 433.57 | 41.89% |
| Fluoxetine | 345.79 | 48.70% |
| Trazodone Hydrochloride | 414.36 | 62.74% |
| Mirtazapine | 265.36 | 116.66% |
| Sertraline | 342.69 | 65.42% |
| Citalopram | 405.30 | 146.52% |
| Vortioxetine | 298.45 | 75.56% |
| Vonoprazan fumarate | 461.46 | 80.02% |
| Pantoprazole Sodium | 423.38 | 52.02% |
| Esomeprazole | 345.42 | 42.00% |
| Duloxetine | 333.88 | 69.12% |
| Clomipramine | 351.32 | 69.80% |
| Nimodipine | 418.44 | 58.96% |
| Atorvastatin | 558.65 | 66.20% |
| Dronedarone | 593.20 | 29.96% |
| Captopril | 217.28 | 64.28% |
| Valsartan | 435.53 | 106.92% |
| Bosentan | 551.61 | 88.71% |
| darusentan | 410.42 | 89.46% |
| Irbesartan | 428.54 | 72.60% |
| Clonidine Hydrochloride | 266.56 | 91.45% |
| rivaroxaban | 435.88 | 84.36% |
| Candesartan | 440.46 | 85.17% |
| Lovastatin | 404.54 | 57.96% |
| Nebivolo | 441.90 | 64.48% |
| Carvedilol | 406.47 | 13.69% |
| Telmisartan | 514.62 | 10.36% |
| Quinidine | 324.42 | 87.01% |
| Cilostazol | 369.47 | 26.84% |
| Ticagrelor | 286.29 | 8.48% |
| Simvastatin | 418.57 | 31.58% |
| Amlodipine | 408.88 | 63.43% |
| Ambrisentan | 378.42 | 100.22% |
| Anagrelide | 256.09 | 94.06% |
| Disopyramide | 339.47 | 99.65% |
| Metoprolol Tartrate | 684.82 | 95.25% |
| Apixaban | 459.51 | 102.39% |
| Rosuvastatin Calcium | 1001.14 | 86.91% |
| Nisodipine | 388.41 | 29.76% |
| Warfarin Sodium | 330.31 | 135.57% |
| Azilsartan | 456.46 | 89.52% |
| Clevidipine butyrate | 456.32 | 93.83% |
| Trandolapril | 430.54 | 96.88% |
| losartan | 422.91 | 79.79% |
| Felodipine | 384.25 | 24.52% |
| Osimertinib | 499.61 | 98.72% |
| Enasidenib | 473.37 | 100.00% |
| Kaempferiae Acid | 462.36 | 79.13% |
| Resveratrol | 228.25 | 79.74% |
| Emodin | 270.24 | 10.48% |
| Berberine | 336.36 | 81.38% |
| Silybin | 482.44 | 58.71% |
| Arctiin | 534.55 | 32.23% |
| Alkannin | 288.31 | 5.96% |
| Matrine | 248.37 | 89.20% |
| Rhizoma Curcumae | 266.42 | 26.94% |
| Rubusoside | 642.73 | 82.51% |
| Wogonoside | 460.39 | 85.26% |
| Olanzapine | 312.44 | 80.36% |
| Baicalin | 446.36 | 64.47% |
| Artemether | 298.38 | 35.08% |
| Guttiferin | 396.43 | 59.67% |
| Crizotinib | 450.34 | 78.67% |
| Efavirenz | 315.68 | 54.41% |
| Cycloastragenol | 490.72 | 55.94% |
| Glycyrrhetinic Acid | 470.69 | 61.32% |
| Propofol | 178.28 | 104.25% |
| Paeonol | 166.18 | 79.09% |
| Limonin | 470.52 | 20.64% |
| Curculigoside | 466.44 | 81.82% |
| Nodakenin | 408.40 | 101.06% |
| Cyasterone | 520.65 | 102.53% |
| Apigenin | 270.24 | 39.92% |
| Cucurbitacin B | 558.70 | 96.56% |
| Andrographolide | 350.46 | 95.35% |
| Physcion | 284.26 | 94.81% |
| Arctiin | 534.55 | 104.09% |
| Genistein | 274.26 | 70.44% |
| Tetrandrine | 622.75 | 91.44% |
| Artemether | 298.38 | 39.93% |
| Procyanidin | 468.42 | 25.27% |
| Kaempferol | 286.23 | 75.60% |
| Calycosin | 284.26 | 89.99% |
| Myricetin | 318.24 | 2.42% |
| Proanthocyanidins | 594.52 | 58.03% |
| Evonimine | 805.78 | 111.53% |
| Thymol | 150.22 | 109.62% |
| Astragaloside | 784.97 | 116.67% |
| Tiliroside | 594.52 | 31.21% |
| Sophoridine | 248.36 | 110.91% |
| Oxymatrine | 264.36 | 105.65% |
| Genistein | 270.24 | 83.77% |
| Aminophylline | 180.16 | 109.09% |
| Baicalein | 270.24 | 110.51% |
| Schizandrin | 432.51 | 118.45% |
| Piperine | 285.34 | 39.93% |
| Glycyrrhetic acid | 470.69 | 62.44% |
| Deoxyschizandrin | 416.51 | 114.45% |
| Chlorogenic acid | 354.31 | 173.00% |
| Wogonin | 284.26 | 134.24% |
| Lycopene | 536.88 | 53.81% |
| Chrysin | 254.23 | 14.99% |
| Triptolide | 360.40 | 83.51% |
| Vanillylacetone | 194.22 | 171.07% |
| Quercetin | 302.23 | 56.32% |
| Praeruptorin B | 426.46 | 152.35% |
| Schizandrin | 432.51 | 116.91% |
| Gossypol. | 518.56 | 3.42% |
| Naringenin | 272.25 | 103.75% |
| Arctigenin | 372.41 | 56.20% |

153 drugs were purchased from Shanghai Macklin Biotech Co., Ltd., Biochempartner, Meilunbio, Med Chem Express, Perfemiker, Aladdin or Target Mol.

**Supplementary Table 2**. The main pharmacokinetic parameters and their ratio (metabolite/parent ratio) of aumolertinib and HAS-719 in three groups of AAV8–Ctrl mice.

| AAV8–Ctrl Parameters | Aumolertinib | | | HAS-719 | | |
| --- | --- | --- | --- | --- | --- | --- |
|  | Aum | Tel+Aum | Car+Aum | Aum | Tel+Aum | Car+Aum |
| AUC_(0-t)_ (μg/L·h) | 1,797.33±314.94 | 3,230.87±1,317.94* | 1,973.65±611.81 | 831.84±163.19 | 1,231.85±372.97* | 772.28±187.06 |
| AUC_(0-∞)_ (μg/L·h) | 1,853.69±351.87 | 3,808.60±1,715.31* | 1,977.45±611.75 | 834.68±162.92 | 1,461.52±582.30* | 775.56±185.44 |
| t_1/2z_ (h) | 8.21±5.10 | 9.43±4.77 | 3.12±1.79* | 4.10±2.21 | 8.91±5.10 | 4.53±3.69 |
| T_max_ (h) | 1.29±0.49 | 2.43±1.13* | 17.71±10.74** | 2.86±2.48 | 15.43±16.03 | 21.14±7.56*** |
| V_z/F_ (L/kg) | 98.79±56.30 | 68.22±44.59 | 38.62±23.71* | 119.81±66.03 | 154.71±104.14 | 158.28±180.79 |
| CL_z/F_ (L/h/kg) | 8.90±1.64 | 4.98±2.08** | 8.71±2.39 | 19.74±3.52 | 12.30±4.06** | 21.62±5.01 |
| C_max_(μg/L) | 176.89±59.92 | 163.36±65.58 | 99.84±27.53** | 55.04±8.67 | 50.66±18.82 | 40.11±9.45** |

metabolite/parent ratio

| Group Parameters | Aum | Tel+Aum | Car+Aum |
| --- | --- | --- | --- |
| AUC_(0–t)_ (μg/L·h) | 0.46±0.09 | 0.38±0.12 | 0.39±0.09 |
| AUC_(0–∞)_ (μg/L·h) | 0.45±0.09 | 0.38±0.15 | 0.39±0.09 |
| t_1/2z_ (h) | 0.50±0.27 | 0.94±0.54 | 1.45±1.18 |
| T_max_ (h) | 2.22±1.93 | 6.35±6.60 | 1.19±0.43 |
| V_z/F_ (L/kg) | 1.21±0.67 | 2.27±1.53 | 4.10±4.68 |
| CL_z/F_ (L/h/kg) | 2.22±0.40 | 2.47±0.81 | 2.48±0.58 |
| C_max_ (μg/L) | 0.31±0.05 | 0.31±0.12 | 0.40±0.09* |

*P < 0.05, **P < 0.01, ***P <0.001 in comparison with the control group. AUC: area under the blood concentration-time curve; t1/2z: elimination half time; Tmax : peak time; Vz/F: apparent volume of distribution; CLz/F: blood clearance; Cmax : maximum blood concentration.

**Supplementary Table 3**. The main pharmacokinetic parameters and their ratio (metabolite/parent ratio) of aumolertinib and HAS-719 in three groups of AAV8–CYP3A4*1 mice.

| AAV8–CYP3A4*1 Parameters | Aumolertinib | | | HAS-719 | | |
| --- | --- | --- | --- | --- | --- | --- |
|  | Aum | Tel+Aum | Car+Aum | Aum | Tel+Aum | Car+Aum |
| AUC_(0-t)_ (μg/L·h) | 1,369.00±462.80 | 2,890.74±1,426.46* | 1,733.06±545.95 | 905.88±375.90 | 429.91±178.00* | 663.56±198.24 |
| AUC_(0-∞)_ (μg/L·h) | 1,388.11±456.46 | 3,710.03±2,673.60 | 1,973.18±564.02 | 908.18±374.69 | 572.38±354.11 | 669.24±196.18 |
| t_1/2z_ (h) | 7.89±2.76 | 5.75±3.22 | 9.63±8.92 | 3.80±2.94 | 14.62±18.23 | 5.34±2.61 |
| T_max_ (h) | 1.64±1.18 | 16.29±14.06* | 11.57±11.66 | 10.71±9.53 | 14.29±13.44 | 17.86±10.53 |
| V_z/F_ (L/kg) | 164.89±124.88 | 49.86±33.64* | 104.52±73.56 | 113.97±104.36 | 773.46±1,086.45 | 203.88±121.79 |
| CL_z/F_ (L/h/kg) | 13.28±6.56 | 6.59±4.36* | 8.72±2.51 | 20.10±7.69 | 35.20±14.99* | 25.43±6.17 |
| C_max_(μg/L) | 95.44±26.37 | 154.38±62.70 | 96.55±39.00 | 47.06±20.43 | 23.01±11.89* | 34.97±15.02 |

metabolite/parent ratio

| Group Parameters | Aum | Tel+Aum | Car+Aum |
| --- | --- | --- | --- |
| AUC_(0–t)_ (μg/L·h) | 0.50±0.21 | 0.15±0.06** | 0.38±0.11 |
| AUC_(0–∞)_ (μg/L·h) | 0.49±0.20 | 0.15±0.10** | 0.34±0.10 |
| t_1/2z_ (h) | 0.48±0.37 | 2.54±3.17 | 0.56±0.27 |
| T_max_ (h) | 2.24±1.99 | 0.88±0.83 | 1.54±0.91 |
| V_z/F_ (L/kg) | 0.72±0.66 | 15.51±21.79 | 1.95±1.17* |
| CL_z/F_ (L/h/kg) | 1.63±0.62 | 5.34±2.27** | 2.92±0.71** |
| C_max_ (μg/L) | 0.44±0.19 | 0.15±0.08** | 0.36±0.16 |

*P < 0.05, **P < 0.01, ***P <0.001 in comparison with the control group. AUC: area under the blood concentration-time curve; t1/2z: elimination half time; Tmax : peak time; Vz/F: apparent volume of distribution; CLz/F: blood clearance; Cmax : maximum blood concentration.

**Supplementary Table 4**. The main pharmacokinetic parameters and their ratio (metabolite/parent ratio) of aumolertinib and HAS-719 in three groups of AAV8–CYP3A4*12 mice.

| AAV8–CYP3A4*12 Parameters | Aumolertinib | | | HAS-719 | | |
| --- | --- | --- | --- | --- | --- | --- |
|  | Aum | Tel+Aum | Car+Aum | Aum | Tel+Aum | Car+Aum |
| AUC_(0-t)_ (μg/L·h) | 3,302.93±1,151.29 | 4,456.23±1,744.40 | 2,476.75±1,160.83 | 1,430.86±656.22 | 1,607.78±429.43 | 849.79±292.79 |
| AUC_(0-∞)_ (μg/L·h) | 3,461.89±1,184.39 | 8,016.28±6,329.77 | 2,510.78±1,154.08 | 1,551.65±644.42 | 1,804.18±372.75 | 874.56±302.97* |
| t_1/2z_ (h) | 11.95±5.16 | 29.15±28.88 | 6.44±2.34* | 11.21±10.86 | 14.77±10.12 | 7.77±3.56 |
| T_max_ (h) | 5.14±8.32 | 9.43±10.18 | 8.00±7.57 | 11.71±11.51 | 12.86±10.57 | 16.00±10.07 |
| V_z/F_ (L/kg) | 83.16±30.50 | 90.34±85.03 | 70.39±38.65 | 168.60±160.90 | 209.40±169.74 | 219.12±96.58 |
| CL_z/F_ (L/h/kg) | 5.23±2.26 | 3.08±2.06 | 7.45±2.84 | 12.25±5.81 | 9.22±2.03 | 20.03±6.26* |
| C_max_(μg/L) | 193.69±48.84 | 185.59±74.37 | 143.27±102.47 | 75.45±24.05 | 69.37±22.45 | 40.04±11.66** |

metabolite/parent ratio

| Group Parameters | Aum | Tel+Aum | Car+Aum |
| --- | --- | --- | --- |
| AUC_(0–t)_ (μg/L·h) | 0.43±0.20 | 0.36±0.10 | 0.34±0.12 |
| AUC_(0–∞)_ (μg/L·h) | 0.45±0.19 | 0.23±0.05* | 0.35±0.12 |
| t_1/2z_ (h) | 0.94±0.91 | 0.51±0.35 | 1.21±0.55 |
| T_max_ (h) | 2.28±2.24 | 1.36±1.12 | 2.00±1.26 |
| V_z/F_ (L/kg) | 2.03±1.93 | 2.32±1.88 | 3.11±1.37 |
| CL_z/F_ (L/h/kg) | 2.34±1.11 | 2.99±0.66 | 2.69±0.84 |
| C_max_ (μg/L) | 0.39±0.12 | 0.37±0.12 | 0.28±0.08 |

*P < 0.05, **P < 0.01, ***P <0.001 in comparison with the control group. AUC: area under the blood concentration-time curve; t1/2z: elimination half time; Tmax : peak time; Vz/F: apparent volume of distribution; CLz/F: blood clearance; Cmax : maximum blood concentration.
